# Clinical relevance of histologic subtypes in locally advanced esophageal carcinoma treated with pre-operative chemoradiotherapy: Experience of a monographic oncologic centre

**DOI:** 10.1371/journal.pone.0184737

**Published:** 2017-09-20

**Authors:** Maria Saigí, Marc Oliva, Luisa Aliste, Mariona Calvo, Gloria Hormigo, Òlbia Serra, Anna Boladeras, Leandre Farran, Javier Robles, Gloria Creus, Ma José Paúles, Joan B. Gornals, Eugenia de Lama, Josep Ma Borràs, Núria Sala, Maica Galán

**Affiliations:** 1 Department of Medical Oncology, Institut Català Oncologia (ICO), IDIBELL, L’Hospitalet de Llobregat, Barcelona, Spain; 2 Gastroesophageal Tumours Functional Unit (UTEG), Institut Català d’Oncologia- Hospital Universitari de Bellvitge- IDIBELL, L'Hospitalet de Llobregat, Barcelona, Spain; 3 Evaluation Unit, Cancer Plan, Department of Health, L’Hospitalet de Llobregat, Barcelona, Spain; 4 Department of Radiation Oncology, Institut Català Oncologia (ICO), IDIBELL, L’Hospitalet de Llobregat, Barcelona, Spain; 5 Digestive Surgery Department, Hospital Universitari de Bellvitge, IDIBELL, L’Hospitalet de Llobregat, Barcelona, Spain; 6 Nuclear Medecine Department, Institut del Diagnòstic Imatge (IDI), Hospital Universitari Bellvitge, IDIBELL, L’Hospitalet de Llobregat, Barcelona, Spain; 7 Clinical Nutrition Unit, Hospital Universitari de Bellvitge, IDIBELL, L’Hospitalet de Llobregat, Barcelona, Spain; 8 Pathology Department, Hospital Universitari de Bellvitge, IDIBELL, L’Hospitalet de Llobregat, Barcelona, Spain; 9 Gastroenterology Department, Hospital Universitari de Bellvitge, IDIBELL, L’Hospitalet de Llobregat, Barcelona, Spain; 10 Radiology Department, Hospital Universitari de Bellvitge, IDIBELL, L’Hospitalet de Llobregat, Barcelona, Spain; 11 Department of Clinical Sciences, University of Barcelona, IDIBELL, L’Hospitalet de Llobregat, Barcelona, Spain; 12 Unit of Nutrition and Cancer, Cancer Epidemiology Research Program and Translational Research Laboratory, Institut Català d’Oncologia (ICO)-IDIBELL, L’Hospitalet de Llobregat, Barcelona, Spain; University Medical Center of Princeton at Plainsboro, UNITED STATES

## Abstract

**Background:**

Locally advanced esophageal carcinoma (LAEC) represents less than 30% of all diagnosed esophageal carcinoma worldwide. The standard of care for resectable tumours consists of preoperative chemoradiotherapy (CRT) followed by surgery. Despite the curative intent, the prognosis is still poor mainly due to relapse. A multidisciplinary approach is required in order to optimize the therapeutic strategy and follow-up. Differences in outcomes between the two main histological subtypes, adenocarcinoma (ADC) and squamous cell carcinoma (SCC), have been reported. Nevertheless, the heterogeneity in trials design and data available have hampered the achievement of clear conclusions. The purpose of this study is to report the outcomes from a cohort of patients with LAEC treated with a multidisciplinary approach and to remark the differences observed between the two main histologic subtypes and their clinical implications.

**Methods:**

We retrospectively reviewed 100 patients diagnosed with LAEC that were treated with preoperative CRT at our institution and integrated centres. Histopathological characteristics and toxicities during treatment were recorded. Patterns of recurrence at the first relapse were analysed. Survival curves were plotted using the Kaplan Meier method and multivariate Cox proportional hazards models were used.

**Results:**

Among the patients who received preoperative CRT, 83% underwent surgery. The median overall survival (mOS) was 31.7 months, 26.9 months for ADC and 45.5 for SCC (p-value = 0.33). In the multivariate Cox regression analysis, ypN+ was the only factor that negatively influenced in OS (OR = 4.1, p-value = 0.022). Patterns of recurrence differed according to histologic subtype. Distant relapse was more frequent in ADC (62%), whereas locoregional relapse was higher in SCC (50%) (p-value = 0.027). Second line therapeutic strategies could be offered to 50% of those patients who relapsed.

**Conclusions:**

Differences in outcomes and recurrence pattern could be observed between the two main histologic subtypes of LAEC. A better molecular characterization, adapted therapeutic regimens and follow up strategies should be adopted in order to improve survival of these patients.

## Introduction

The incidence of esophageal carcinoma in Europe is around 4.5 cases/100.000 habitants per year, causing more than 400,000 deaths annually [1]. The aggressive behaviour of the disease confers a poor prognosis, with a five-year overall survival (OS) that rarely exceeds 40% in locally advanced tumours, and less than 5% in metastatic disease [2].

Over the last few decades, esophageal carcinoma incidence and mortality have varied substantially across the world, with important differences between the two main histological subtypes, squamous cell carcinoma (SCC) and adenocarcinoma (ADC). Although SCC accounts for about 90% of cases worldwide, it has declined substantially in some regions the last few years, whereas the incidence of ADC has progressively increased. Because the pathogenesis of both subtypes is different, the distribution pattern is changing and varies across the regions depending mostly on the life-style habits. Alcohol and tobacco consumption are associated with SCC whereas the main risk factors for ADC are high fat diet, obesity and gastroesophageal reflux disease (GERD) [3–5].

Locally advanced esophageal carcinoma (LAEC) comprises those tumours that invade through the muscular layer and involve other adjacent structures or lymph nodes (T3-4N0 and T1-4aN1, M0) [6]. It represents a potentially curable disease but prognosis is limited mainly due to relapse [7]. Several clinical trials attempted to determine whether the preoperative CRT is superior to other therapeutic approaches in this setting. Different staging techniques, and heterogeneous trial designs and data, are some of the reasons that explain the lack of strong evidence about which strategy we might follow. Recently, a number of single-institution trials have been performed demonstrating the benefit of concurrent chemoradiotherapy (CRT) followed by surgery in LAEC. It achieves higher rates of complete tumour resection (R0) without increasing surgical mortality, better locoregional tumour response (ypTN), and micrometastatic disease control that enhances better survival [8–11]. Despite the differences observed in response rate and prognosis according to histologic subtype, there are no consistent data to support that histology is a predictive biomarker of CRT in esophageal carcinoma [12].

The main purpose of this retrospective study is to report the outcomes and prognosis in a cohort of patients with LAEC treated with concurrent CRT followed by surgery, in a monographic oncologic institution, and to describe patterns of recurrence, assessing the influence of histologic subtypes.

## Methods

### Study population

This study was approved by the Institutional Review Board and Ethics Committee from Hospital Universitari de Bellvitge, and recorded data was anonymized for analysis. In this retrospective study, we reviewed 100 consecutive patients diagnosed with LAEC treated at the Catalan Institute of Oncology (ICO), Bellvitge University Hospital (HUB) and integrated centres between 2005 and 2014. Eligible patients for this study had histological or cytological confirmation of esophageal carcinoma. Staging assessment confirmed locally advanced disease (T3-4N0 and T1-4aN1, M0). Carcinomas of the esophago-gastric junction (EGJ) Siewert 1 and Siewert 2 (Siewert 2, only until 2012), were also included. All patients were reviewed in the gastro-esophageal multidisciplinary tumour board, a multidisciplinary committee composed by different healthcare professionals that comprises radiologists, surgeons, oncologists, gastroenterologists, pathologists, nutritionists, and specialised nurses. Those patients considered candidate to receive concurrent CRT followed by surgery were included in the study.

The staging assessment included gastric fibroscopy, endoscopic ultrasonography (EUS) when confirmation of locoregional lymph nodes involvement was needed, and a bronchoscopy in all supracarinal tumours or in case of airway infiltration suspicion. A baseline positron emission tomography scan (PET CT) was performed in all cases to exclude distant metastasis. The final stage was established according to the International Union against Cancer (UICC) and tumour—node—metastasis (TNM) classification (AJCC/UICC 7th Edition) [6].

### Therapeutic approach and follow-up

Patients were candidates to receive preoperative chemoradiotherapy (CRT) with of Cisplatin (80 mg/m^2^/d) and infusional 5-Fluoracil (1000 mg/m^2^/d) doublet on days 1 to 4. Carboplatin was used in case of renal function impairment, hearing loss, or elderly. Intensity-modulated radiotherapy dose of 45 or 50,4 Gy in 1,8 Grays/fraction was administered depending on the centre protocol.

The overall response rate was evaluated by PET CT four weeks after completing RT. In case of tumour response or stable disease, surgery was offered to all fit patients with operable tumours.

Surgical techniques were performed depending on tumour location and surgical risk was assessed for every patient. McKeown for supracarinal tumours (transthoracic esophaguectomy plus neck gastroplasty), Ivor-Lewis for infracarinal tumours (esophaguectomy plus intrathoracic gastroplasty) or transhiatal esofaguectomy in case of frail patients or comorbidities.

All G3-4 toxicities during the CRT treatment, as well as postoperative mortality within the first 30 days, were recorded.

The pathology report described histologic subtype, grade of differentiation, vascular, perineural and lymphatic invasion, surgical margins and tumoral regression assessment according to Mandard classification [13,14]. A microscopic margin of less than 1 mm was considered as positive, and proximal and distal macroscopic margins of at least 2 cm beyond gross tumour were required for considering negative surgical margins. The ypTNM (AJCC/UICC 7th Edition, 2010) was evaluated in order to know the downstaging after preoperative CRT.

Follow-up controls were conducted regularly after therapy. A thoracic—abdominal CT scan was performed annually since the date of surgery, until 5 years follow-up. Pattern of recurrence: locoregional, distant, or synchronic locoregional and distant were recorded for the two main histologic subtypes (ADC and SCC).

### Statistics

Data was collected retrospectively. Chi-square test (*X*^*2*^) was used for categorical variables comparison. Median follow-up was calculated from the starting treatment date until the last follow-up for surviving patients. Kaplan—Meier method was used to plot overall survival (OS) and progression free survival (PFS) and log-rank test was used to estimate statistical differences, with 95% confidence intervals (CI). Univariate and multivariate analyses were performed by the Cox proportional hazard regression model to assess the prognostic value of different histopathological variables. A multiple regression analysis for recurrence was also performed. Two-tailed *p*-value less than 0.05 was established to assume significant differences. All analyses were carried out using the SPSS v.21.0 (IBM Corp., Armonk, NY, USA).

## Results

### Characteristics of patients

Between 2005 and 2014, 100 patients were included in the study. Clinico-pathological characteristics and treatment received are summarized in Table 1. Median age was 61 years old (range 34–78). Most of them were male (93%) and current or former smokers (77%). Most frequent histology was ADC (53%), followed by SCC (43%). 82% of tumours were located in esophagus whereas 18% in the EGJ. 91% of patients presented ≥ cT3-4 and 88% had nodal involvement (cN+) at diagnosis.

**Table 1:** Characteristics of patients.

	All Patients N = 100 (100%)
**Age—years (range)**	
Median	61 (34–78)
**Sex—n° (%)**	
Male	93 (93)
Female	7 (7)
**Tobacco habit—n° (%)**	
Never	14 (14)
Former	38 (38)
Current	39 (39)
Unknown	9 (9)
**Alcohol habit—n° (%)**	43 (43)
Never	21 (21)
Former	27 (27)
Current	9 (9)
Unknown	
**Performance Status—n° (%)**	
PS0	3 (3)
PS1	86 (86)
PS2	3 (3)
Unknown	8 (8)
**Barrett Esophaegus—n° (%)**	
Yes	18 (18)
No	82 (82)
**Histology—n(%)**	
Adenocarcinoma	53 (53)
Squamous Cell Carcinoma	43 (43)
Undifferentiated	2 (2)
*Not determined*	2 (2)
**Location—n° (%)**	
Esophagus	
Upper	7 (7)
Middle	35 (35)
Lower	40 (40)
OGJ—Siewert	
Siewert 1	13 (13)
Siewert 2	5(5)
**cT—n° (%)**	
Tx	1 (1)
T2	8 (8)
T3	70 (70)
T4a	19 (19)
T4b	2 (2)
**cN—n° (%)**	
Nx	2 (2)
N0	10 (10)
N+	88 (88)

All patients received preoperative concurrent CRT. The median cycles of CT were 2. Cisplatin-5-Fluoracil was the most used regimen (83%) and it was well balanced between the two main histologic subtypes (86% of SCC, and 81% of ADC). 76% of patients received 45 Gy of RT dose, whereas 18% of patients, 50.4Gy, depending on the centre protocol. Treatment characteristics in the all cohort (N = 100) and according to the two main histologic subtypes (SCC, N = 43 and ADC, N = 53) is indicated in Table 2. The overall response rate was 19% complete response (CR), and 55% partial response (PR). 83% of patients were able to undergo radical surgery. Grade 3/4 toxicity events during preoperative CRT occurred in 18% of patients, mostly due to G3 neutropenia (5% of patients), G3 mucositis (4%), G3 esophagitis (4%), followed by G3 emesis (3%) and G3 febrile neutropenia (2%). Deaths due to post-operative complications (within 30 days) occurred in 2 patients (2.4%).

**Table 2:** Treatment received by all cohort of patients and according to main histologic subtypes (SCC and ADC).

Treatment	All Patients N = 100 (100%)	SCC N = 43 (100%)	ADC N = 53 (100%)
**NA CT—n° (%)**	83 (83%)		
CDDP-5FU	9 (9)	37 (86%)	43 (81%)
CBDCA-5FU	2 (2)	1 (2)	8 (15)
CBDCA-Taxol	3 (3)	1(2)	1 (2)
Other	3 (3)	2(5)	0 (0)
Unknown		2 (5)	1 (2)
**N° of cycles (CT)**			
Median (range)	2 (1–4)	2 (1–4)	2 (1–3)
**RT (Gy)—n° (%)**			
45	76 (76%)	31 (72%)	41 (79%)
50,4	18 (18)	10 (23)	8 (15)
Other	6 (6)	2 (2)	4 (6)
**Median dose (Gy)**	45 (40–65)	45 (45–63)	45 (40–65)
**Surgery—n° (%)**			
Yes	83 (83%)	35 (81%)	45 (85%)
No	17 (17)	8 (19)	8 (15)
**Reason for no surgery—n° (%)**	**N = 17**	N = 8	N = 8
Unresectable	11 (64,7%)	5 (63%)	6 (75%)
Patient denial	2 (11,8)	1 (12)	1 (12.5)
Other	4 (23,5)	2 (25)	1 (12.5)

NA, Neo-adjuvant; CDDP, Cisplatin; CBDCA, Carboplatin; 5FU, 5-Fluoracil; CT, chemotherapy; RT, radiotherapy.

### Pathological assessment and outcomes

Among 83 resected tumours, 54% were ADC and 42% SCC. 87% had negative margins (R0). Pathologic complete response (ypCR) was achieved in 24% of the patients (18% of resected ADC (n = 8) and 34% SCC (n = 12), p = 0.12). Lymph node downstaging rate, from positive to negative nodes was 61% (44/72 patients). Tumour pathologic response using Mandard regression score was also recorded. Histopathologic results are summarized in Table 3.

**Table 3:** Pathologic results from patients with resection (surgery).

Pathologic Results	All patients with resection *N = 83*			
**Histology—n° (%)**				
Adenocarcinoma	45 (54%)			
Squamous cell carcinoma	35 (42)			
Undifferentiated	2 (3)			
Unknown	1 (1)			
**Margins**—n°(%)				
R0	72 (87%)			
R1	6 (7)			
Unknown	5 (6)			
**Downstaging T3/4 (n = 77) to Tx/1/2**—n° (%)				
Yes	44 (57%)			
No	33(43)			
**Downstaging cN+ (n = 72) to ypN0**—n° (%)				
Yes	44 (61)			
No	28 (39)			
		**SCC**	**ADC**	***P- value***
**ypCR**—n° (%)	**N = 80**	***N = 35***	***N = 45***	
Yes	20 (24%)	12 (34%)	8 (18%)	0.12
No	60 (76%)	23 (66%)	37 (82%)	

ypCR, pathologic complete response.

At the time of the analysis, median follow-up for surviving patients was 38.4 months (range 8–118). Recurrence rate (RR) at 1^st^ and 3r year were 36% and 43%, respectively.

Median disease-free survival (DFS) was not reached for SCC subgroup, whereas for ADC was 16.5 months (p<0.24).

**Fig 1:**
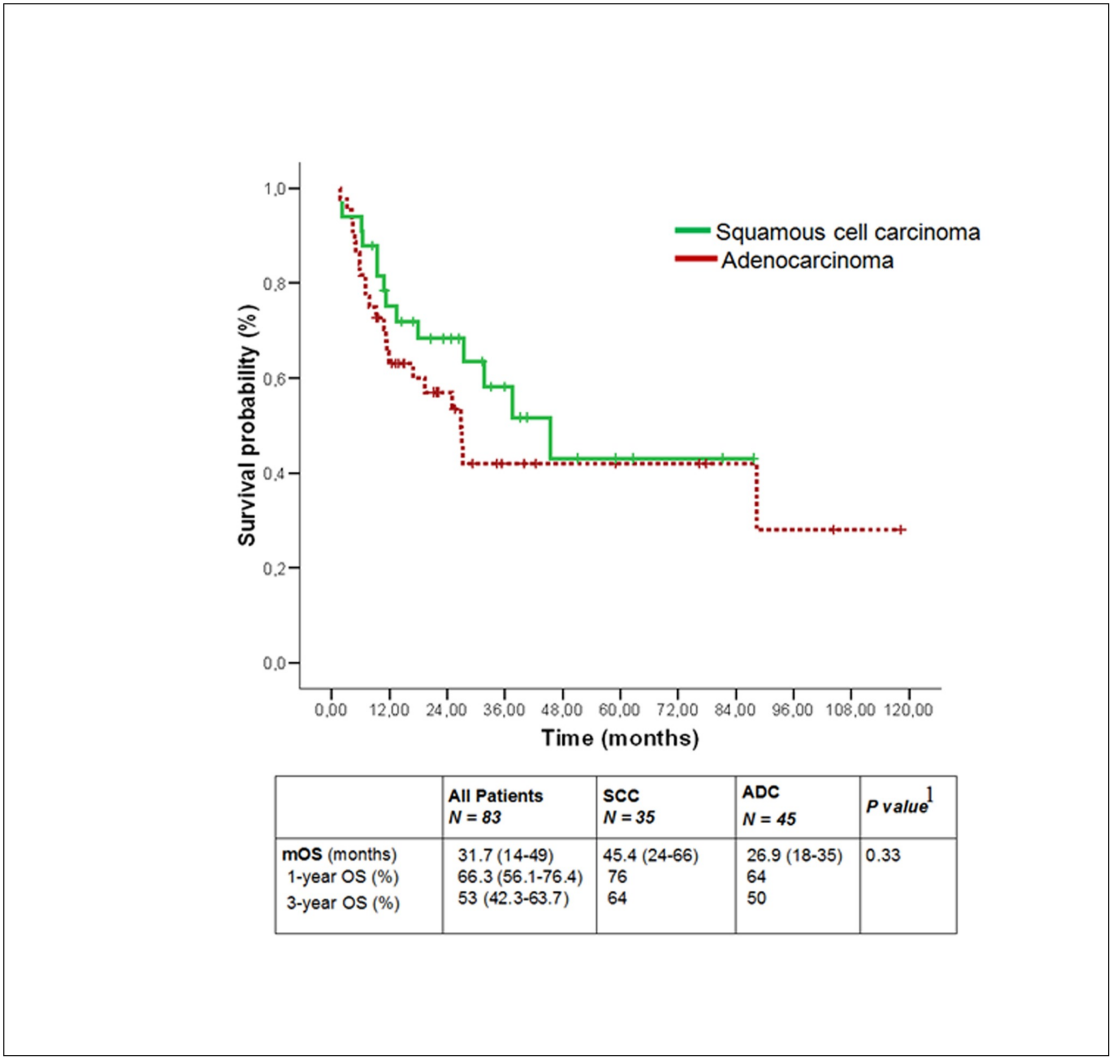
Overall survival (OS) according to histologic subtype.

Median OS was longer for SCC 45.4 m than for ADC 32 m, despite no significant differences were found (p-value = 0.33). Overall survival rates at 1^st^ and 3^rd^ year were 66,3% and 53%, respectively (Fig 1).

In the multivariate analysis of OS adjusted by age, sex, histology, ypCR, and response to NA CRT, ypN+ was an independent prognostic factor for OS (HR = 2.24, 95% CI 1.14–4.38; p-value = 0.019). In the multiple logistic regression analysis for recurrence risk, ypN+ remained as a predictive factor for recurrence (OR = 4.4; 95% CI 1.6–13.1; p-value = 0.022) (Table 4).

**Table 4:** Multiple logistic regression analysis for recurrence in all patients with resection (n = 83).

Variable	OR (CI 95%)	*P- value*
Gender (*male* vs female)	0.00	*0*.*99*
Age (*<60y* vs > 60 years)	0.9 (0.3–2.8)	*0*.*87*
Histology: *ADC* vs SCC	0.7 (0.24–2.12)	*0*.*5*
cN (*cN0* vs cN+)	2.5 (0.24–26.5)	*0*.*43*
ypCR (*non ypCR* vs ypCR)	1.8 (0.42–7.6)	*0*.*43*
ypN (*ypN0* vs ypN+)	4.1 (1.22–13.7)	*0*.*022*

Note: Reference variables in italics.

ADC, adenocarcinoma; SCC, squamous cell carcinoma; ypCR, pathologic complete response; OR, Odds Ratio.

### Pattern of recurrence

The overall recurrence rate (RR) of patients who underwent surgery was 43% (36 patients): 34% of resected SCC (n = 12), and 47% of resected ADC (n = 21). Among all of them, 55% had distant recurrence, being ADC the most frequent histology (72%); 12% had exclusively locoregional relapse, being 75% SCC; the remaining 33% had synchronic locoregional and distant recurrence, p-value = 0.027 (Table 5).

**Table 5:** Recurrence rate and patterns of recurrence according to histologic subtype.

**Recurrence Rate**	**All Patients *N = 83***	**SCC *N = 35***	**ADC *N = 45***	
**Recurrence Rate—n (%)**				
Overall	36–43%	12–34%	21–47%	
1^st^ year	30–36%	9–26%	18–40%	
3^rd^ year	36–43%	12–34%	21–47%	
**Site of recurrence**	***N = 33 (100%)***	**SCC *N = 12***	**ADC*N = 21***	***P-value***
**Locorregional (LR)**—n (%)	8 (24%)	6 (75%)	2(25%)	0.027
**Distant (D)**—n (%)	18 (55%)	5 (28%)	13 (72%)	
**Synchronic LR+D**—n (%)	7 (21%)	1 (14%)	6 (86%)	

Among the patients who relapsed (n = 36), 50% were candidates to receive further treatment. Two patients presented a single metastatic site at progression after surgery. Both were treated with curative intention. The first one, who had a SCC, underwent salvage surgery for the metastasis in the brain. Unfortunately, he died due to post-surgical complications. The other one, diagnosed with ADC, received concurrent CRT (for locoregional lymph nodes relapse) and the survival after relapse improved by 16 months. The rest of patients (86%) were eligible for palliative chemotherapy (n = 15) and palliative external RT (n = 1) (Table 6).

**Table 6:** Treatment at recurrence.

Treatment at first recurrence	*N = 18 (100%)*
Radiotherapy—n (%)	1 (6)
Chemo-RT—n (%)	1 (6)
Salvage surgery—n (%)	1 (6)
Palliative chemotherapy—n (%)	15 (83)
Docetaxel	7 (47)
Cisplatin plus 5-fluoracyl	3 (20)
Carboplatin plus 5-fluoracyl	2 (13)
Others	1 (7)
*Unknown*	2 (13)

Among this group of patients, 90% had performance status ≤1. Median time on treatment was 2.1 months (range <1-5m) and 38% of patients received subsequent lines of chemotherapy. Median OS from recurrence date was 3 months (CI 95% 0.5–5.6). 6.5m (4.4–8.5) for patients who received any treatment at recurrence compared with 1.6m (1–2.3) for those who received best supportive care (p-value = 0.026).

## Discussion

The two main histologic subtypes of esophageal carcinoma are SCC and ADC. They represent two distinct diseases with different epidemiology and prognosis. Preoperative treatment in both histologies is clearly indicated in operable patients. In a recent clinical trial (CROSS trial, NTR487), preoperative chemo-radiotherapy showed significantly higher response rate in SCC compared to ADC, but no significant differences in OS could be demonstrated [15].

The multidisciplinary approach delivered to our patients lead to an optimal individual staging which determined preoperative decision making. Nowadays, several clinical guidelines recommend the involvement of multidisciplinary teams in the management of oncologic patients due to the benefits evidenced on outcomes [16]. One of the goals successfully achieved by our multidisciplinary tumour board has been to standardize and optimize therapeutic strategies for all patients with positive benefit on outcomes over the last few years [17,18].

Our cohort comprised 21 patients (21%) with T4 staging at diagnosis and 88% cN+, conferring a more aggressive disease according to the TNM classification 7^th^ Edition AJCC [6]. Among the patients who underwent surgery after concurrent CRT (83%), ypCR was achieved in 24% of cases, with a higher proportion of SCC subtype. cN+ downstaging was achieved in 61% of patients, thus ypN+ resulted as an independent prognostic factor for recurrence disease and low survival in the multivariate Cox Regression analysis, consistent with previous reports [19,20].

Regardless the improvement of surgical techniques and radiotherapy constraints, half of the patients with LAEC will relapse, mostly within the first 3 years during the follow up. In our series, distant recurrence was more prevalent for ADC, whereas SCC tended to relapse loco-regionally (p-value = 0.032), probably due to a higher potential metastatic risk associated with ADC.

Surveillance after treatment is performed routinely for both histologic subtypes and includes image test, and biomarker test with CA 19–9 and CEA, only for adenocarcinomas. The effort to find potential predictive biomarkers in this setting will help us to identify not only which patients are at higher risk of relapse, but also to characterize recurrence patterns which leads to adapt follow up strategies [21].

Differences in OS according to histologic subtype were more favourable to SCC compared to ADC (45m vs 26m) despite not being statistically significant. Differences in OS and recurrence pattern according to histologic subtypes have been previously reported [22–24] but data are not consistent because no phase III randomized clinical trials have properly addressed this issue.

### Second line strategies

Further therapeutic strategies in case of relapse or refractoriness are scarce and there is no consensus on the optimal second-line regimen because of the lack of efficient therapies. Furthermore, current data on second-line treatment recommendations are based on relatively small studies in a highly-selected cohort of patients. However, approximately 50% of patients who relapse are potential candidates to receive second line treatment, mostly those who preserve a good performance status [25]. Local therapies such as RT or surgery represent efficient options that offer more aggressive approach when disease progresses in a single metastatic site. In our series, we observed 11% of oligoprogressions among patients who relapsed. Despite its low prevalence, there is a growing interest in many cancer types due to its potential benefit in OS when curative-intention options can be offered [26]. Larger cohorts of patients addressing this issue should be developed in order to stablish more conclusive statements.

### Identifying biomarkers

Esophageal carcinoma represents a heterogeneous disease with different outcomes and response grade to conventional therapies. The attempt to establish molecular subtypes based on genetic and molecular alterations tries to find potential predictive and prognostic biomarkers in order to investigate the role of tailored therapies to improve the current treatment. [27–29].

Amplification of *ERBB2*, which encodes for the HER2 protein is present almost in 7 to 34% of esophagogastric ADC. The TOGA phase III trial, demonstrated improvement in OS with the addition of Trastuzumab to conventional first line chemotherapy in patients with metastatic/recurrent HER2–expressing gastric and EGJ cancer [30]. This was the first study to incorporate a biologic agent into the treatment of upper gastrointestinal cancer. Its implications for locally advanced disease are currently being investigated in the Radiation Therapy Oncology Group 1010 trial (NCT01196390), which incorporates trastuzumab with a CROSS-like regimen for HER2-overexpressing esophageal ADC.

Two large-scale studies from the ICGC (International Cancer Genome Consortium) and TCGA (The Cancer Genome Atlas) have been recently published with the aim to better characterize esophageal cancer at the molecular level [31, 32]. They demonstrate that cancers that develop in the same location can vary greatly at this level. The mutational profile of esophageal SCC closely resembles that of squamous cell carcinomas from other tissues, and the genomic alterations profile of esophageal ADC is closely similar to the CIN (chromosomal instability) variant of gastric cancer.

## Conclusions

Despite recent improvements in staging techniques and supportive management of LAEC its prognosis is still poor, mostly due to early recurrences. The optimal management of these patients should involve a multidisciplinary team of healthcare professionals in order to improve therapeutic results.

Because ADC and SCC have distinct predisposing risk factors and clinic-pathologic characteristics, histology-specific assessment is relevant in terms of prevention and therapeutic management. The different outcomes evidenced in our retrospective cohort between the two main histologic subtypes has not been directly assessed in randomized clinical trials but the differences observed might lead to improve the follow-up approach in this setting. In addition, an optimal molecular characterization might help us to better understand pathogenesis of the disease and will contribute to develop further therapeutic strategies. Efforts to validate new predictive biomarkers, together with the histopathological subtype, would help to identify which patients are at higher risk of relapse in our current clinical practise, in order to offer them rationally designed follow up and therapeutic strategies.
